# Tough Gelatin Hydrogel for Tissue Engineering

**DOI:** 10.1002/advs.202301665

**Published:** 2023-06-23

**Authors:** Ximin Yuan, Zhou Zhu, Pengcheng Xia, Zhenjia Wang, Xiao Zhao, Xiao Jiang, Tianming Wang, Qing Gao, Jie Xu, Debin Shan, Bin Guo, Qingqiang Yao, Yong He

**Affiliations:** ^1^ State Key Laboratory of Advanced Welding and Joining Harbin Institute of Technology Harbin 150001 P. R. China; ^2^ National Innovation Center for Advanced Medical Devices Shenzhen 457001 P. R. China; ^3^ State Key Laboratory of Oral Diseases National Clinical Research Center for Oral Diseases West China Hospital of Stomatology Chengdu 610041 P. R. China; ^4^ Institute of Digital Medicine Nanjing First Hospital Nanjing Medical University Nanjing 210006 P. R. China; ^5^ State Key Laboratory of Fluid Power and Mechatronic Systems School of Mechanical Engineering Zhejiang University Hangzhou 310027 P. R. China; ^6^ Key Laboratory of 3D Printing Process and Equipment of Zhejiang Province College of Mechanical Engineering Zhejiang University Hangzhou 310027 P. R. China; ^7^ Cancer Center Zhejiang University Hangzhou 310058 P. R. China

**Keywords:** gelation hydrogel, tendon regeneration, tissue engineering, tough hydrogel

## Abstract

Tough hydrogel has attracted considerable interest in various fields, however, due to poor biocompatibility, nondegradation, and pronounced compositional differences from natural tissues, it is difficult to be used for tissue regeneration. Here, a gelatin‐based tough hydrogel (GBTH) is proposed to fill this gap. Inspired by human exercise to improve muscle strength, the synergistic effect is utilized to generate highly functional crystalline domains for resisting crack propagation. The GBTH exhibits excellent tensile strength of 6.67 MPa (145‐fold that after untreated gelation). Furthermore, it is directly sutured to a ruptured tendon of adult rabbits due to its pronounced toughness and biocompatibility, self‐degradability in vivo, and similarity to natural tissue components. Ruptured tendons can compensate for mechanotransduction by GBTH and stimulate tendon differentiation to quickly return to the initial state, that is, within eight weeks. This strategy provides a new avenue for preparation of highly biocompatible tough hydrogel for tissue regeneration.

## Graphical abstract

Inspired by human muscle strengthening, tough gelatin hydrogel is prepared by directed training and ion‐induced synergistic crystallization. Furthermore, tough gelatin hydrogel can be implanted into the body to repair damaged tendons due to their high toughness and biocompatibility. Tough gelatin hydrogel is expected to be an ideal material for tendon repair.

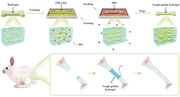

## 1 Introduction

Tough hydrogel has been a major research focus in the past decades due to excellent mechanical properties, and it has been commonly used in soft artificial muscles, tissue engineering scaffolds,^[^ ^1^ ^]^ flexible electronics,^[^ ^2^, ^3^ ^]^ and other applications.

Various methods to produce tough hydrogel have been reported with the objective to improve their mechanical properties, such as freeze‐casting,^[^ ^4^, ^5^ ^]^ mechanical training in the air or water,^[^ ^6^, ^7^, ^8^, ^9^ ^]^ and adding micro‐nano materials.^[^ ^10^, ^11^, ^12^ ^]^ Freeze casting‐assisted salting out can be used to produce highly anisotropic hydrogel with properties comparable to natural tendons, including micron‐scale honeycomb‐like pore walls that include interconnected nanofibrous webs. Repeated mechanical loading allows physically cross‐linked hydrogel to rearrange along the loading direction, thereby improving its inherent mechanical properties, allowing for various potential uses. Double‐network tough hydrogel is produced by introducing rigid structural materials, and the stress concentration and crack propagation are solved based on the principle of sacrificial bonds. Tough hydrogel has been fabricated through various strategies, however, those prepared through the methods mentioned above have limitations such as poor biocompatibility, difficulty in auto‐degradation in vivo, and considerable differences from natural tissue components, thus such materials are still distinctly different from human skin,^[^ ^13^, ^14^ ^]^ brain,^[^ ^15^, ^16^ ^]^ and other organs.^[^ ^17^, ^18^ ^]^ Therefore, these tough hydrogels cannot be implanted into the body for medical applications such as tissue engineering^[^ ^19^, ^20^, ^21^ ^]^ and drug delivery.^[^ ^22^, ^23^, ^24^ ^]^

To address these shortcomings, numerous novel strategies were proposed. A previous study used a tough double‐network hydrogel produced from alginate, polyacrylamide, Ca^2+^, and chitosan, and the hydrogel can be applied directly to a damaged tendon as a bonding layer to repair the tendon.^[^ ^25^ ^]^ In a different study, a self‐healing hydrogel was prepared from substances such as gelatin‐methacryloyl (GelMA), hyaluronic acid, and polycaprolactone, which can be used as a protective layer to repair damaged tendons in rats.^[^ ^26^ ^]^ Further, calcium silicate nanowires and alginate were previously used to obtain composite hydrogel with bio‐inspired enhanced structures. Composite hydrogel significantly promotes the regeneration of tissues from bone to tendons in the body, especially in fibrocartilage transition tissues.^[^ ^27^ ^]^ Tough hydrogel has been fabricated through different strategies, however, these methods are relatively complex exhibit poor consistency, and require various materials, which complicates clinical application. Therefore, tough hydrogel with components meeting practical needs is required.

Natural tissues such as tendons and ligaments require a certain degree of toughness to support weight and perform multiple functions.^[^ ^28^, ^29^ ^]^ Healthy or damaged tendons can achieve self‐reinforcing or rapid recovery through repeated mechanical loading. This mechanical training stimulates tendons to reshape their structure and develop increased strength and direction.^[^ ^7^ ^]^ Compared with randomly distributed structures, such highly ordered structures can withstand higher more axial forces.^[^ ^7^, ^8^ ^]^

Here, we propose a novel and convenient strategy inspired by human muscle training. Based on the method of salt solution‐assisted stretching, a gelatin‐based tough hydrogel (GBTH) was produced merely by placing gelatin‐based hydrogel in a salt solution, applying axial cyclic stress several times at room temperature, immersing in phosphate‐buffered saline (PBS), and repeating the process several times. No additional chemical modifications were made. Owing to its excellent mechanical properties (tensile strength up to 6.67 MPa, that is,145‐fold higher than in the initial state), biocompatibility, auto‐degradability in vivo, and similarity to natural tissue components, GBTH can be sutured directly to ruptured tendons of adult rabbits. By compensating for mechanotransduction and stimulating tendon differentiation, the ruptured tendon can quickly recover within eight weeks. This strategy provides a new avenue for tissue regeneration engineering, which is expected to outcompete traditional methods of treating tendon injuries in clinical practice.

## 2 Results and Discussion

### 2.1 Design of Tough Gelatin‐Based Hydrogel

**Figure 1:**
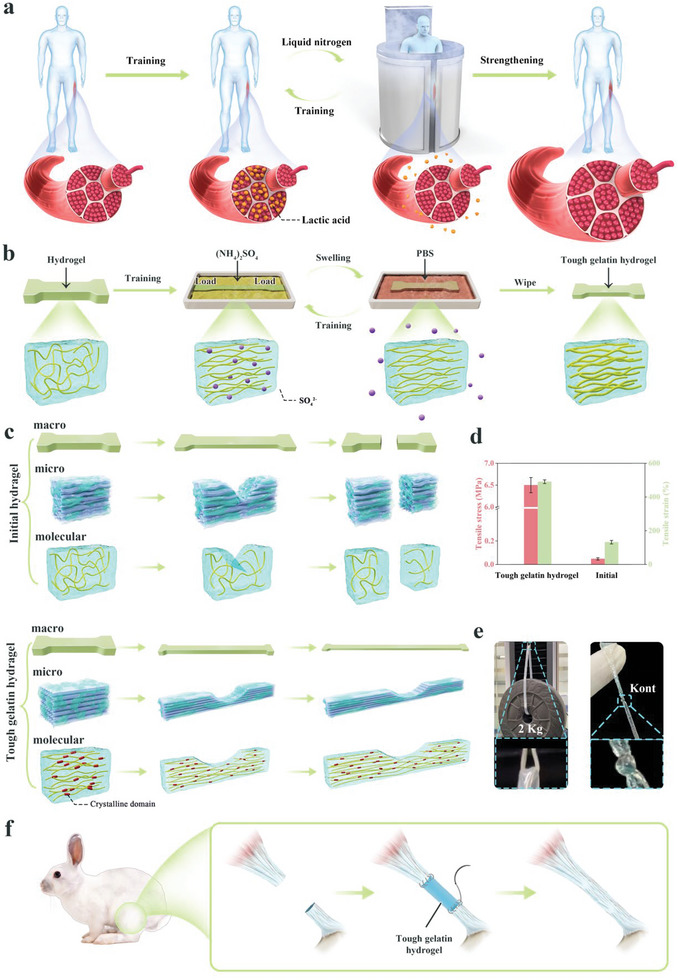
Schematic diagram of tough hydrogel preparation. a) Schematic diagram of human muscle strengthening. b) Schematic diagram of high biocompatibility and highly tough hydrogel preparation. c) Schematic diagram of the mechanism of hydrogel intensification. d) Mechanical properties of tough hydrogel. e) Highly tough hydrogel used to lift weights. f) Schematic diagram of in vivo implantation to repair tendons.

Natural self‐training and reinforcement processes include human self‐training during which the strength of muscle fibers is increased, however, this frequently produces large amounts of lactic acid. Excessive lactic acid accumulation can have adverse effects, and liquid nitrogen rapid cooling has been used by professional athletes to quickly release accumulated lactic acid during rapid‐recovery training (**Figure** **1**a).

We developed a convenient way to obtain tough hydrogel with high biocompatibility, single components, and biomimetic structure without additional chemical modifications or additives. GelMA hydrogel was considered a model system owing to its high biocompatibility. The hydrogel was placed in a salt solution for simple mechanical training (Figure 1b). The molecular chains inside the hydrogel thus re‐arranged in order along the direction of the axial load, and crystallinity increased. According to the Hofmeister effect,^[^ ^30^ ^]^ under the influence of SO_4_^2−^, the pre‐concentrated hydrogel chains strongly self‐aggregated and separated from the original homogeneous phase. The phase separation of the hydrogel developed over time, until structure complexity and crystallinity were sufficient. Next, the trained hydrogel was immersed in PBS, thus expelling SO_4_^2−^ from the hydrogel which is replaced by water molecules, thus ensuring biocompatibility. The PBS treatment resembled the liquid nitrogen treatment after human exercise, and SO_4_^2−^ is equivalent to lactic acid produced during human exercise. After several treatment repetitions of training in salt solution and immersion in PBS, the binding effect of the internal chains of the hydrogel became stronger (similar to human self‐training to strengthen muscle fibers).

More importantly, the arrangement of the chains became highly ordered, and crystallinity increased. This increased crystallinity during the salt solution training process strengthened the hydrogel molecular chain and improved its inherent elasticity. Crystalline domains can act as rigid, highly functional crosslinkers, thus they can delay the rupture of a single molecular chain through crack pinning, making the original hydrogel tough (Figure 1c). Our strategy can increase the strength of ordinary hydrogel 145‐fold, and tensile strength reached 6.67 MPa. The GBTH was able to withstand a weight of 2 kg (1000‐fold its own weight), and it did not break when tightened by a knot (Figure 1d,e). Further, GBTH can be directly sutured to a ruptured tendon in adult rabbits and achieve rapid recovery of the damaged tendon to the initial state within eight weeks, mainly because GBTH can compensate for the mechanical conduction function of the tendon and activate tendon differentiation (Figure 1f).

### 2.2 Formation of Homogeneous Structure

**Figure 2:**
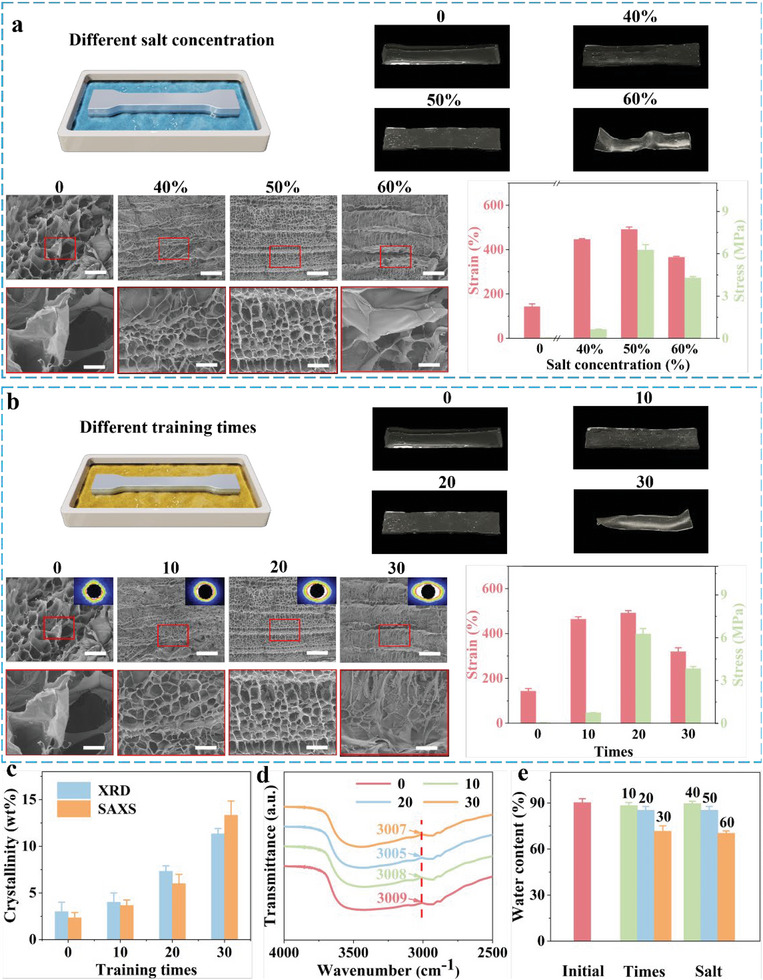
Mechanical properties of gelatin‐based tough hydrogel. a) Mechanical properties of hydrogel as a function of salt concentration. b) Mechanical properties of hydrogel as a function of the number of training cycles. c) Crystallinity of the hydrogel as a function of the number of training cycles. d) FTIR characterization of hydrogel. e) The water content of hydrogel with different treatments. The insets show the corresponding SAXS patterns of hydrogel. Scale bars: initial is 100 µm, further enlargement is 20 µm.

We show mechanical properties of GBTH and explain its toughness mechanism (**Figure** **2**). Salt concentration and the number of training cycles were considered two main parameters (Figure 2a,b, Figures S1 and S2, Supporting Information, and keep another unchanged when discussing one variable). The mechanical properties of hydrogel improved with increasing salt concentration (Figure 2a, the number of training times is 20 times); however, when the salt concentration exceeded 50%, the mechanical properties of hydrogel started to deteriorate. Macroscopic imaging showed that the hydrogel shrunk to different degrees, which was attributed to spontaneous aggregation of molecular chains caused by the Hofmeister effect.^[^ ^31^ ^]^ Corresponding SEM imaging indicated that low salt concentrations tended to weaken the aggregation of molecular chains, resulting in large pore size of honeycomb pores inside the hydrogel and poor uniformity. Furthermore, honeycomb pores inside the hydrogel tended to be highly arranged and uniform in pore size when the salt concentration approached 50%, whereas at >50%, the molecular chains arrange disorderly, leading to the destruction of the internal spatial structure and deterioration of the mechanical properties of hydrogel. Thus, the optimal salt concentration was 50%.

After cyclic stretching in salt solution several times, the hydrogel was immediately immersed in PBS solution for 1 min as a complete mechanical training. The performance of hydrogel increased with the number of training cycles; however, at >20 cycles, the performance started to decrease (Figure 2b). Macroscopic images showed that when the training cycles reached 30, the hydrogel was severely deformed and difficult to recover. SEM images indicated that at ten training cycles the honeycomb pores inside the hydrogel started to appear aligned (consistent with SAXS imaging), however, the aperture uniformity was poor. The number of training sessions was further increased, and the honeycomb pores in the hydrogel tended to be highly arranged. The SAXS plot showed high crystallinity (the order degree of molecular chain arrangement), and pore size was uniform. At this time, the mechanical properties of hydrogel reached the best value. However, when the number training cycles exceeded 20, the homogeneous honeycomb structure of the hydrogel was destroyed. In addition, XRD and DSC characterization methods were performed to more accurately describe crystallinity (Figure 2c, Figures S3 and S4, Supporting Information). Although more than 20 training cycles led to higher crystallinity, the toughness of the material deteriorated when the crystallinity exceeded a certain threshold, leading to reduced mechanical quality of the material. Thus, high crystallinity should not be considered the main objective.

Apart from the crystal structure, strong hydrogen bond interactions between the hydrogel polymer chains improve mechanical properties of hydrogel. Harnessing the strength of hydrogen bonds in hydrogel is considered to be an effective way to improve mechanical properties, where hydrogen bonds are dynamic crosslinking sites in hydrogel, providing “sacrificial domains” for mechanical energy dissipation.^[^ ^32^, ^33^ ^]^ FTIR was used to characterize the hydrogen bond interaction between hydrogel polymer chains (Figure 2d). The broad band at 3009 cm^−1^ corresponded to the stretching vibration of CH_3_. As the number of training cycles increased from 10 to 20, $\upsilon_{{\mathrm{CH}}_{3}}$increased from 3008 to 3005 cm^−1^, indicating a gradual increase in the strength of hydrogen‐bonding interactions (a blue shift occurred). However, from 20 to 30 training cycles, $\upsilon_{{\mathrm{CH}}_{3}}$ decreased from 3005 to 3007 cm^−1^, indicating a gradual weakening (red‐shifted) in the strength of hydrogen‐bonding interactions. This result was consistent with the mechanical properties of the hydrogel, demonstrating that more than 20 stretches reduce the mechanical quality. High water content of hydrogel an important characteristic, and too many training cycles or too high salt concentration will lead to excessive water loss and reduce the quality (Figure 2e). Therefore, the optimal experimental parameters were 20 training cycles and 50% salt concentration, on which the following discussion is based.

### 2.3 Strengthening While Toughening

**Figure 3:**
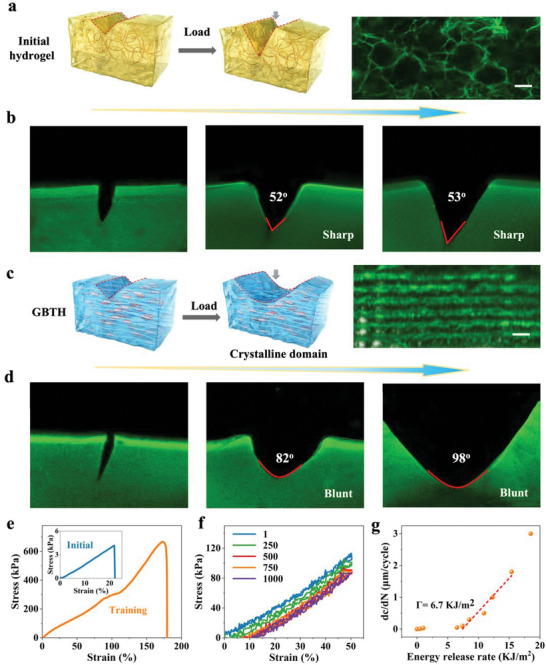
Strengthening while toughening. a) Toughening mechanisms of initial hydrogel. b) Pre‐stretched notched initial hydrogel where the crack is perpendicular to the longitudinal direction of the homogeneous honeycomb. c) Toughening mechanisms of gelatin‐based tough hydrogel. d) Pre‐stretched notched gelatin‐based tough hydrogel where the crack is perpendicular to the longitudinal direction of homogeneous honeycomb. e) Stress–strain curve of notched hydrogel. f) Fatigue resistance of notched hydrogel. g) Energy release rate of notched hydrogel. Scale bars is 100 µm.

In situ confocal laser scanning microscopy further explained the high fatigue threshold mechanism of the GBTH (**Figure** **3**). The initial hydrogel was isotropic, with no directional structure and poor pore size uniformity. More importantly, they had no crystalline domains with versatility to pin crack propagation (Figure 3a). The notch had a sharp and acute angle during the pre‐stretching process, which was unable to passivate cracks, suggesting that this was a typical isotropic material (a red line indicates the sharp notch topography in Figure 3b).

In contrast to the initial hydrogel, GBTH exhibited obvious anisotropy. Training induced anisotropy in the hydrogel, resulting in a directional arrangement of honeycomb structures and reducing the pore size to increase its density to strengthen the hydrogel (Figure 3c). According to the energy dissipation mechanism, the energy dissipation rate in the process of notch fracture was increased to make the hydrogel tougher, and crystalline domains acting as rigid, high‐functional performance crosslinkers were generated during crystallization, enabling them to pin cracks and delay crack propagation. The GBTH showed a gradual failure form during notched pre‐stretching, and no crack propagation perpendicular to the stretching direction was observed. Further, the notch exhibited a rounded obtuse angle, suggesting that the hydrogel can passivate cracks (a red line indicates the blunt notch topography). Even the notch close to 100° did not break the hydrogel, indicating that the GBTH was highly resistant (Figure 3d, Figure S5, Supporting Information).

Notably, due to the induced anisotropy, the notched GBTH exhibited excellent mechanical properties when stretched parallel to the alignment direction: tensile strength of 667 kPa, and fracture toughness 78.5 KJ m^−2^ were increased 159‐fold and 1189‐fold, respectively, compared to the initial notched hydrogel (Figure 3e). To assess the fatigue crack propagation resistance of the GBTH, 1000 cycles of 50% deformation were performed (Figure 3f, Figure S6, Supporting Information and Movie S1, Supporting Information), and the GBTH maintained excellent mechanical properties and exhibited a high fatigue threshold of 6.7 KJ m^−2^ (Figure 3g).

In a word, the strengthening mechanism is mainly due to the formation of hydrogen bonds and crystalline domains leading to a pronounced increase in structural density, while the toughening mechanism was due to the formation of solvent‐induced structural anisotropy of the hydrogel, passivation of cracks by crystalline domains, and energy dissipation. During structural development at multiple scales, the tensile strength and elongation at break of the hydrogel increased simultaneously.

### 2.4 Fatigue Resistance

**Figure 4:**
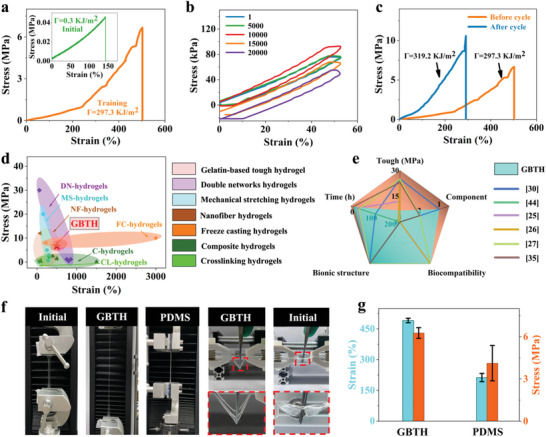
Properties of gelatin‐based tough hydrogel. a) Stress–strain curves of gelatin‐based tough hydrogel. The inset is the stress–strain curve of a hydrogel in the initial state. b) Fatigue resistance of gelatin‐based tough hydrogel. c) Self‐reinforcing properties of gelatin‐based tough hydrogel. d) Ashby image of tensile strength and elongation at break of gelatin‐based tough hydrogel. e) Comparison of tough hydrogel with another representative reported reinforced hydrogel. f) Optical images of hydrogel. g) Comparison of mechanical properties of gelatin‐based tough hydrogel and representative silica‐based PDMS.

To confirm the superior muscle‐like properties of the GBTH, cyclic fatigue tests were conducted. The mechanical properties of the initial hydrogel were qualitatively improved after muscle‐like training, and the strain, stress, and fracture energy values were increased 3.5‐, 146‐, and 991‐fold, respectively (**Figure** **4**a). Further, cyclic axial stretching of the GBTH was carried out to assess its fatigue resistance and test its applicability for practical use. The hydrogel showed some hysteresis, which was due to the presence of reversible sacrificial structures (mainly hydrogen bonds). Even after 20 000 axial cycles, it maintained a similar state to that after the first cycle, demonstrating excellent fatigue resistance of the GBTH (Figure 4b, Movie S2, Supporting Information). There may be two reasons for the downward shift of the curve with increase of the number of cycles: one may be the mechanism of the universal testing machine, and the other may be a certain plastic deformation of the hydrogel due to a long time and a large number of cyclic stretching. Surprisingly, the mechanical properties of the hydrogel improved significantly, even after 20 000 cycles of fatigue and proper training, which was consistent with the results of the previous training method, suggesting superior self‐reinforcement of the GBTH (Figure 4c). These results suggest that fatigue is not a concern regarding practical applicability (although the strain was slightly reduced, it was sufficient for many application scenarios).

Comparisons with currently used methods for strengthening hydrogel (freeze‐casting,^[^ ^30^, ^34^ ^]^ nanofiber,^[^ ^35^, ^36^, ^37^, ^38^ ^]^ mechanical stretching,^[^ ^7^, ^8^, ^9^, ^39^ ^]^ double‐network,^[^ ^40^, ^41^ ^]^ composite,^[^ ^42^, ^43^, ^44^ ^]^ and crosslinking hydrogel^[^ ^45^, ^46^, ^47^ ^]^ showed the advantages of our processing method (Table S1, Supporting Information). Although our approach was not optimal for enhancing the stress‐strain of hydrogel, it allows preparing a tough hydrogel with a single component, highly biocompatibility, and biomimetic structure through a simple and convenient strategy. More importantly, this hydrogel has clinical transformation advantages and is expected to be industrialized (Figure 4d,e).

In addition, the outstanding mechanical properties of the GBTH can be observed directly in pictures (Figure 4f, Movie S3, Supporting Information). With our strategy, it can be stretched several times over the initial state of the hydrogel. Furthermore, we compared the mechanical properties of GBTH with those of PDMS (a common silicone gel). Its mechanical properties exceeded those of PDMS, with its deformation and tensile strength being approximately twice those of PDMS (Figure 4g). Of note, our strategy has made it possible to produce a tough hydrogel film that can withstand a sharp tweezer thrust vertically, which by far exceeds the resistance ability of the initial gelatin‐based hydrogel.

### 2.5 Simulation of Functional Compensation

**Figure 5:**
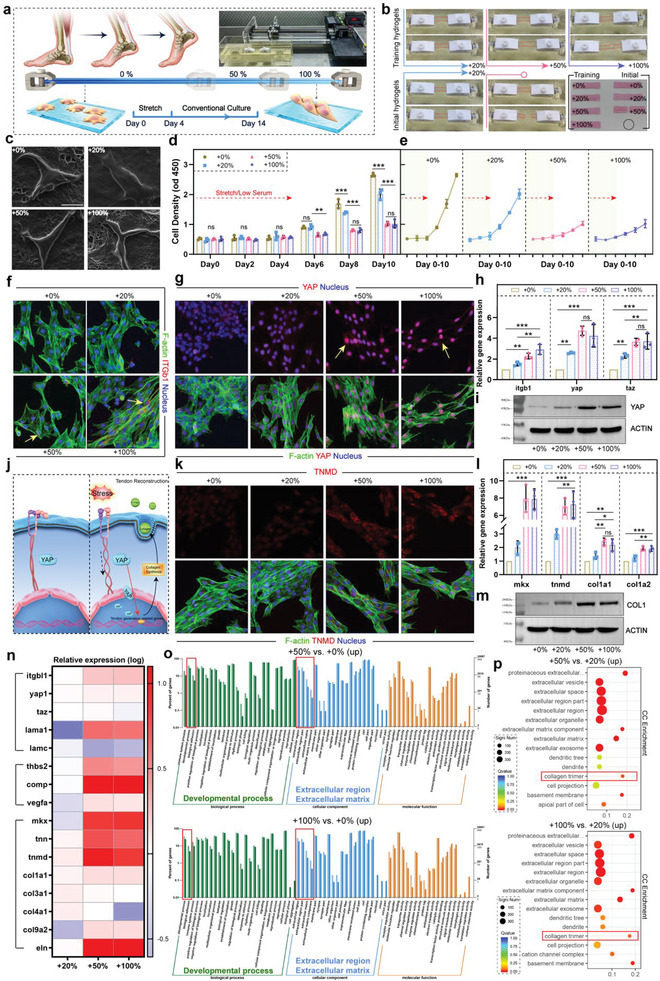
a) Introduction to muscle contraction simulation, 0–100% represents the ratio of elongation and deformation. b) The operational feasibility of the initial hydrogel and the trained hydrogel on the simulator. c) Cell morphology under SEM. d,e) TPSC proliferation behavior. **p < 0.01; ***p < 0.001, *n* = 3. f) Immunofluorescence staining of ITG*β* (red), F‐actin (green), and the nucleus (blue). g) Immunofluorescence staining of YAP (red), F‐actin (green), and the nucleus (blue). h) Expression of gene of *itgb1*, *yap*, and *taz*. **p < 0.01; ***p < 0.001, *n* = 3. i) Protein expression level of YAP. j) Tough hydrogel compensating for the mechanotransduction capacity of tendon. k) Immunofluorescence staining of TNMD (red), F‐actin (green), and the nucleus (blue). l) Expression of gene of *mkx*, *tnmd, col1a1*, and *col1a2*. *p = 0.033; **p < 0.01; ***p < 0.001, *n* = 3. m) Protein expression level of COL1. n) Heatmap of several related genes by transcriptome analysis. o) Results of GO analysis. p) Results of cellular component enrichment. All scale bars indicate 30 µm.

When muscles contract, the tendons follow the reciprocating movement and bear the complex and changeable tension. Therefore, in the treatment of injured tendons, in addition to the simple cell growth scaffold, the repair material must exhibit reliable elasticity, maintaining the force conduction of the tissue and prevent contracture of the tendon.^[^ ^48^ ^]^ Considering their muscle‐like properties, we assessed whether the training tough hydrogel can serve as temporary functional compensation for injured tendons. As shown in **Figure** **5**a, the simulation of functional compensation of the tough hydrogel relied on a self‐developed muscle contraction simulator, performed in a typical staged culture as reported previously.^[^ ^49^ ^]^ Compared with tissues such as nerves or blood vessels, muscle tissue has higher stress sensitivity thresholds and requires sufficient stress stimulation to activate its physiological functions. Thus, the operational feasibility of the initial hydrogel and the trained hydrogel on the simulator were verified first. As shown in Figure 5b, the trained hydrogel was able to maintain its own structural integrity and length stability after several stretching cycles with elongation ratios ranging from 0% to 100%. In contrast, the initial hydrogel group only withstood ≈20% elongation deformation and fractured during further stretching, thus making it difficult to apply sufficient stress to the cells. Therefore, the trained hydrogel group was finally included in the study of functional compensation simulation and activation thresholds of tendons.

To examine the viability of cells under different degrees of muscle contraction simulation, cell morphology and proliferative capacity experiments were performed. Although tendon mesenchymal stem cells (TPSCs) showed good survival morphology in the 0–100% groups (Figure 5c), their proliferation varied between groups. In the low serum environment, TPSCs under different degrees of cycling simulation did not show any significant proliferation behavior (Figure 5d), whereas after restoration with normal serum, the 0% and 20% groups with no muscle contraction simulation or less contraction significantly proliferated, especially the 0% group, which showed a typical proliferation curve. Correspondingly, cells in the 50% and 100% experimental groups proliferated more slowly from day 4 to day 10 (Figure 5d,e). This trend may be due to the difference in TPSC differentiation in the 50% and 100% groups, because the ability of mesenchymal stem cells to divide into terminal cells is correspondingly weakened or even lost.^[^ ^50^ ^]^

The key proteins of mechanosensation and response on TPSCs were stained to explore whether the tough hydrogel could partially compensate the force‐conduction function of tendon, which able to reach the threshold activating tendon differentiation. Integrins (ITGs) localized on the cell membrane are major stress receptors.^[^ ^51^ ^]^ After four days of muscle contraction simulation group, ITG*β* on the cell membrane was activated to varying degrees (red fluorescence), and the 100% group was the most obvious (yellow arrow in Figure 5f). Furthermore, as shown in Figure 5g, muscle simulation on tough hydrogel also effectively increased the content of Yes‐associated protein (YAP), which was crucial for cellular mechanical stress perception, while a tendency of YAP moving into the nucleus was observed in the 50% and 100% treatments (yellow arrows). Combined with the localization of TAZ shown in Figure S12, Supporting Information, which is a binding carrier for YAP to enter the nucleus to exert its effects,^[^ ^52^ ^]^ TPSCs in the 50% and 100% groups were force‐activated. Gene and protein expression levels further confirmed the above conclusion that the tough hydrogel can conduct simulated muscle afterburning stimulation, especially the 50% and 100% groups (Figure 5h,i and Figure S7, Supporting Information). Therefore, as a bio‐friendly matrix, tough hydrogel may compensate for the stress function of the damaged tendon, maintain mechanosensitivity of tenocytes, and provide a basis for the repair of tendon injuries by stem cells such as TPSCs (Figure 5j).

We further assessed the performance of tough hydrogel reaching the threshold of promoting tendon differentiation of TPSCs based on the delivery of stress stimuli. On day 14, as shown in Figure 5k–m and Figure S7, Supporting Information, regardless of tenomodulin (TNMD) and Homeobox protein Mohawk (Mkx) which promoted tendon differentiation, or the collagen (Col1) mainly been produced to repair tendon,^[^ ^53^ ^]^ their expression levels were the highest in the 50% and 100% groups, with no significant difference between them. However, although already activating the expression of stress‐related proteins, it seemed that the 20% elongation of the tough hydrogel could not effectively promote the tendon differentiation of TPSCs. Here, transcriptome analyses were used to further confirm the relationship between the amount of deformation of the tough hydrogel and the threshold for inducing differentiation. As shown in Figure 5n, all groups of TPSCs at Day 14 no longer showed significant mechanosensing activity as simulated muscle contraction was terminated. However, the respective genes exerting positive effects on tendon repair such as tenascin family, col1a, and elastin (eln) were highly expressed in the 50% and 100% groups, while their expression in the 20% group remained on the normal level. Besides, the results of Gene Ontology (GO) again confirmed that by applying a stretch of 50% or more of the deformation amount, the differentiation process of the stem cells adhered on the tough hydrogel could be promoted as well as the reconstruction of their extracellular matrix (collagen) (Figure 5o), which is the central requirement of tendon regeneration.^[^ ^54^ ^]^ Regarding muscle contraction simulation below the activation threshold, the collagen production activity of cells in the 20% group was significantly less than that in the 50% and 100% groups (Figure 5p), confirming that they did not enter an efficient tendon repair process. Similarly, with the recoverable deformation limit performed only about 20%, it may be speculated that the initial hydrogel did not have the ability of effective mechanical conduction to promote tendon repair.

### 2.6 Reconstruction of Tendons after Injury

**Figure 6:**
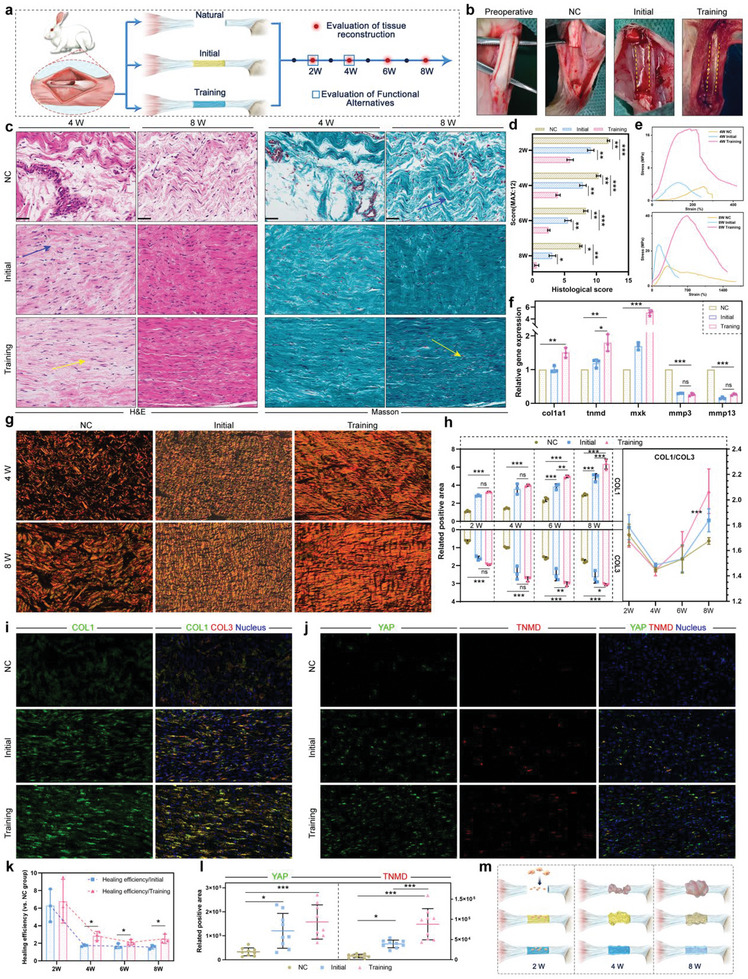
a) Introduction to in vivo tendon repair experiments. b) Intraoperative images. c) H&E and Masson staining of tendon tissues at four and eight weeks. Blue arrows indicate curved fibers, yellow arrows indicate densely aligned fibers, *n* = 3. d) Pathological scoring based on H&E and Masson staining. *p < 0.05; **p < 0.01; ***p < 0.001, *n* = 3. e) Tensile strength of the repaired tendon at four and eight weeks. f) Expression of gene of *col1a1, tnmd, mkx*, *mmp3*, and *mmp13* in tendon tissue at eight weeks. *p = 0.0107; **p < 0.01; ***p < 0.001, *n* = 3. g,h) Polarized observation results and statistics of Sirius red staining. *p = 0.0313; **p < 0.01; ***p < 0.001. *n* = 3. i) Tendon immunofluorescence staining of COL3 (red), COL1 (green), and the nucleus (blue). j) Tendon immunofluorescence staining of YAP (green), TNMD (red), and the nucleus (blue). k) Healing efficiency of training and initial hydrogel (vs NC). *p < 0.05, *n* = 3. l) Statistics of tendon immunofluorescence staining of YAP and TNMD. *p < 0.05; ***p < 0.001, *n* = 9. m) Different stages of tendon reconstruction. All scale bars indicate 200 µm.

After confirming the compensative function for the stress transmitted, the tough hydrogel was used to repair injured Achilles tendons in rabbits. Correspondingly, although not fully qualified as a stress transfer bearer, the initial GelMA acted as a cell scaffold, thus the initial hydrogel was also included in the in vivo study. First, the tough hydrogel was proved to have good biosafety in vivo (Figure S8, Supporting Information). GBTH is biodegradable in vivo, and compared with untrained hydrogels that are rapidly degraded (<14 days), GBTH has a significantly more vital anti‐digestive ability (expected to last for 1 to 2 months), which can provide stable mechanical stress conduction and cell growth templates for tendon repair (Figure S9, Supporting Information). Then as shown in **Figure** **6**a, to provide a comprehensive assessment of the condition of tendon repair, a serially tested rabbit tendon injury model was established. First, the injured tendons were replaced with the training tough hydrogel, and initial hydrogel was loaded to replace the injured tendons while the cut Achilles tendon capsule was preserved (Figure 6b).

The reparation of the tissue microstructure of the tendon, especially the reconstruction of aligned dense collagen fibers, is vital for recovery.^[^ ^54^ ^]^ In the early stage of repair (two weeks), increased amounts of collagen fibers were observed in the damaged area in the hydrogel treatment, compared to the controls, with more regular fibers in the training hydrogel group (Figure S10, Supporting Information). After two more weeks, although the amount of collagen increased in the hydrogel repair groups, the morphology differed, where the newly formed fibers in the training hydrogel group showed aligned linear arrangement (similar to normal tendon collagen morphology), while that in the initial hydrogel group occurred wavily (blue arrow in Figure 6c). This difference in repair effects continued for eight weeks after surgery, when the tissue morphology of the tough hydrogel group after training showed almost no pathological difference from normal tissue (Figure 6c,d). Further, benefitting from this muscle‐like physiological repair with tough hydrogel, the strength of the new tendon was 2–4‐fold stronger than these new tissues in other groups (Figure 6e and Figure S11, Supporting Information). Interestingly, although the initial hydrogel had already shown collagen regeneration at four weeks, its function did not appear to have sufficiently matured to provide better mechanical strength compared to untreated tendons (Figure 6c,e). Further detection of gene expression levels confirmed the above trend (Figure 6f), in which the tendon‐positive *mkx*, *tnmd*, and *col1a1* genes were up‐regulated in the tissues of the training group. At the same time, the expression level of some matrix metalloproteinase was reduced in all hydrogel groups, which has a damaging effect on extracellular matrix collagen,^[^ ^55^ ^]^ thus even the initial hydrogel showed a certain repair ability at eight weeks (Figure 6c–f).

To further assess the beneficial effects of tough hydrogel on injured tendons, newly formed collagen fibers were examined. For tendon reconstruction, type I collagen (COL1) and type III collagen (COL3) may be produced, of which COL1 shows better functional recovery effects.^[^ ^56^ ^]^ As shown in Figure 6g and Figure S12, Supporting Information, the training group showed the most pronounced collagen staining in Sirius Red‐stained polarized observation images. Then, continuous monitoring quantification of collagen production revealed differences between the training, initial, and natural groups (Figure 6h). During the initial period of tendon recovery (0–4 weeks COL1 and COL3 were increased in the hydrogel‐loaded groups and in the controls, and the numbers of fibers (rather than structure) did not differ between groups. At later stages, the superiority of the tough hydrogel became more evident, as COL1 and COL3 regenerated significantly faster than the other groups. More importantly, during the repair process after four weeks, level of COL1 in the training group increased more rapidly than those of COL3 (increased ratio of COL1/COL3), which was crucial for physiological healing of the tendons. Moreover, the above trend was confirmed by specific immunofluorescence staining images of COL1 and COL3 (Figure 6i). As shown in Figure 6k, the repair efficiency based on collagen quantification also showed the temporal characteristics of tough hydrogel treatment of injured tendons. Specifically, the repair efficiency at the early stage of the injured tendon was higher due to the scaffolding function of the hydrogel. Then, owning to the outstanding mechanical properties as well as mechanical conductivity, the tough hydrogel was able to compensate for the missing tendon function and effectively stimulated stem cell differentiation. This positive effect was also shown in the immunofluorescence staining results of YAP and TNMD at four weeks (Figure 6j,l). Other staining images of remine samples could be find in Figure S13, Supporting Information.

Here, it could be speculated that the use of tough hydrogel to healing tendon injuries may be divided into multiple stages (Figure 6m). At the first stage, the hydrogel connected the two ends of the ruptured tendon, thereby providing an important growth substrate and template for migrating repairing stem cells. Therefore, the training and initial hydrogel provided a remarkable recovery boost in the early postoperative period (two weeks). However, with muscle contractions cycles, the initial hydrogel gradually deformed to breaking, failing to support the function of tendon; while the tough hydrogel we prepared still maintained reliable elasticity and continued to act as mechano‐transmitters to activate tendon differentiation continuously owing to their muscle‐like properties (at four weeks). Therefore, in the comparison of the final outcome (at eight weeks), tendon collagen repaired by training tough hydrogel showed multiple advantages in collagen density, proportion, and morphology, which was closest to healthy tissue. The synergistic assistance of the outstanding mechanical properties as well as proper muscle mimicry confirm the advantages of tough hydrogel in tendon repair applications.

### 2.7 Tough Hydrogel Based Liquid Metal Flexible Electrons

**Figure 7:**
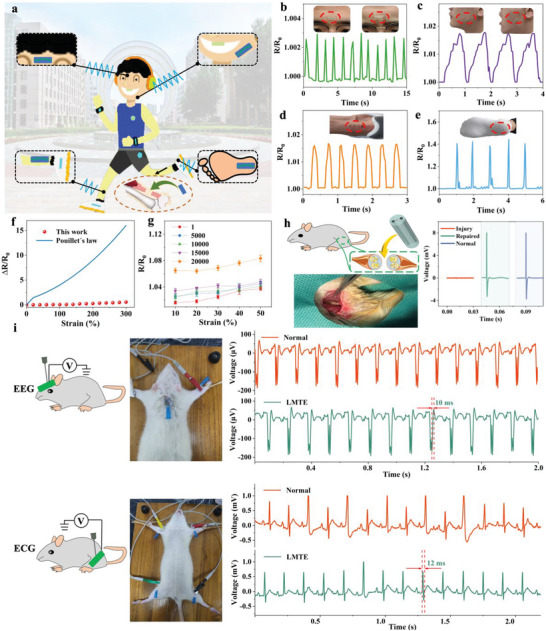
Monitoring capabilities of flexible electron of tough hydrogel. a) Schematic illustration of strain sensing tests on different body parts. Real‐time resistance changes of LMTE when adhered to the middle of the b) forehead, c) cheek, d) heel, and e) sole. f) Relative change in resistance as a function of uniaxial tensile strain of the LMTE. g) Relative change in resistance as a function of cyclic tensile strain of LMTE. h) Implantable LMTE repair functions. i) EEG ECG monitoring capabilities. The insets show the location of the corresponding monitoring.

Tendon injuries are a constant challenge for athletes,^[^ ^57^ ^]^ and our tough hydrogel promise to revolutionize past methods of repairing damaged tendons in the future. Once the tendon is repaired, the athlete can again perform certain movements. Liquid metals were widely used in tissue engineering and biomedical fields because of their high rheology, excellent electrical conductivity, and low toxicity.^[^ ^58^ ^]^ Interestingly, not only do our tough hydrogel repair damaged tendons, but it is also possible to infuse liquid metals into our tough hydrogel to monitor various parts of the human body (**Figure** **7**a).

As partial deformation causes resistance to change, the liquid metal based tough hydrogel electrons (LMTE) can sense the change in resistance and produce corresponding feedback. Surprisingly, when the LMTE was attached to the middle of the forehead, it can sense the changes in human micro‐expressions elicited by emotions. Further, by fixing the LMTE on the cheek, it can feel the deformation caused by a movement process and can then facilitate real‐time monitoring of human health. LMTE can be placed on the heel and sole to analyze human gait during exercise. To sum up, with the excellent performance of LMTE, almost all‐round real‐time monitoring of humans is possible (Figure 7b–e).

The sensor has excellent resistance stability is the key to measure its performance. To this end, the resistance stability of LMTE was developed. The resistance change was smooth even when stretched to 300% (Figure 7f). The lack of strain dependence of this resistance was surprising, with gauge factors well below normal results. This may be mainly due to the strong polar interaction between the hydrogel and Ga_2_O_3_, and the interconnected LMs can adhere to the surface of the hydrogel, which in turn caused the LMs to stretch without failure. Further, the contact area between the LMs particles may increase with stretching, and the initially separated LMs particles were fused together, thus reducing intrinsic resistance. Therefore, LMTE can exhibit excellent resistance stability (Figure S14, Supporting Information). The lifetime of the sensor is a further crucial parameter. LMTE was able to reciprocate axially 20 000 times and still maintain excellent resistance stability, which greatly improves the service life (Figure 7g, Figures S15 and S16, Supporting Information). What's more, with the GBTH as a protective layer, applying stimulation to the LMTE sutured at one end of the nerve, the muscle provided immediate feedback, which was almost the same as that of the normal nerve, suggesting that LMTE initially had the conditions to repair the nerve in vivo (Figure 7h, Figure S17, Supporting Information). This will hopefully solve widespread challenge of repairing damaged nerves in the future. In addition, a tough conductive film has been successfully produced by coating LMs on GBTH, which can be used for EEG and ECG monitoring. The conductive film showed good conductivity and flexibility; the results are similar to those of conventional electrodes, and it can even withstand tweezers without breaking (Figure 7i).

## 3 Conclusion

To conclude, a gelatin‐based tough hydrogel was successfully prepared using salt solution assisted stretching, and its strength was 6.67 MPa, which was 145‐fold higher than that of the initial hydrogel. Furthermore, due to its pronounced toughness and biocompatibility, self‐degradation in vivo, and similarity to natural tissue components, it can be directly sutured to the ruptured tendon of adult rabbits to stimulate tendon differentiation by compensating for mechanical conduction, and quickly return to the initial state within eight weeks. We provide a new strategy for the fabrication of tough hydrogel for tissue engineering and regenerative medicine.

## 4 Experimental Section

### Materials

Phosphate buffered saline (PBS) and Penicillin‐streptomycin liquid (PSL, 100X, reagent grade, with 10 000 units penicillin and 10 000 units streptomycin) were acquired from Zhejiang Jinuo Biomedical Technology Co., Ltd. Lithium phenyl‐2,4,6‐trimethylbenzoylphosphinate (LAP) was obtained from Huaxia Siyin Biotechnology Co., Ltd., Shanghai; Gelatin (G108395 Gelatin, used in microbiology, gel strength ≈ 250 g Bloom) was bought from Aladdin; Liquid metal (Ga_25_In_75_) was acquired from Fanyada Electronic Technology Co., Ltd.; Ammonium sulfate ((NH_4_)_2_SO_4_, AR, 99%) was purchased from Macklin; Electronic fluoride (ETF, MX‐056) was acquired from Suzhou Peng Rui Nano Technology Co., Ltd.; Photoreactive resin HTL (Yellow‐20) was obtained from Chongqing Boston Micro Fabrication (BMF) Technology Co., Ltd.; Polydimethylsiloxane (PDMS, Dow Corning) was from Dow Corning Ethanol (95%) was acquired from Shanghai Lanqing Industrial Co., Ltd. Type‐B gelatin, methacrylic anhydride (MA, ≥94%), sodium hydroxide (NaOH, bioXtra, ≥98%), calcein‐AM (bioreagent, ≥95.0%), and propidium iodide (PI, ≥94%) were purchased from Sigma Aldrich; fetal bovine serum (FBS, Biological Industries, 04‐001‐1A) was acquired from Hyclone, Hong Kong. Deionized (DI) water was prepared by using a laboratory water purification system.

### Fabrication of Hydrogel Solution

First, a 2% PSL solution was prepared using PBS. Next, 1 g of hydrogel, 0.04 g of LAP were added to a pointed bottom centrifuge tube (50 mL), 20 mL of PSL solution was taken and poured into it, then the centrifuge tube was placed in a 60 °C constant temperature water bath until completely dissolved, then it was removed and stored in a 2–8 °C refrigerator for future use.

### Fabrication of Specimen Molds

The specimen mold was manufactured by the S140 printer (BMF). Successfully printed mold is sonicated with ethanol and hydrogen fluoride. Next, PDMS (monomer and curing agent mixed in a ratio of 10:1 and processed by vacuum defoamer) was slowly poured into the cleaned mold and placed in an 80 °C oven to stand for 2 h. Then, an appropriate amount of the prepared hydrogel solution was dropped into the PDMS. Then the mold was placed in a refrigerator at 2–8 °C and was allowed stand for 10 min. Finally, the hydrogel was exposed to blue light for 15 s for curing and the cured hydrogel was removed.

### Hydrogel Strengthening

Two custom‐sized cuboid boxes (without lids) were made into which appropriate amounts of (NH_4_)_2_SO_4_ (w/v 50%) solution and PBS solution were added. The cured hydrogel was completely immersed in the (NH_4_)_2_SO_4_ (w/v 50%) solution, stretched back and forth at a slow speed for a certain number of times, and then immediately immersed in the PBS solution for a period of time. According to this cycle for a certain number of times, a super‐strong hydrogel can be obtained.

### Fabrication of Hydrogel Electronics

The mold was customized by the S140 printer (BMF), the appropriate amount of hydrogel solution was dropped into the mold, put the mold in a 2–8 °C refrigerator and was allowed to stand for 30 min. The hydrogel was then peeled off. Next, the conductive silver cloth was cut and placed in hollow channels at both ends of the hydrogel, glued together, and placed under blue light radiation for 15 s. Finally, liquid metal was injected into the channel of the hydrogel, which was then sealed by dripping the hydrogel at both ends.

### Micromorphology Characterization

In order to characterize the micro‐nano structure of the hydrogel after mechanical training, the hydrogel specimen needs to be immersed in deionized water for 24 h before using the Scientz‐18ND (Ningbo Xinzhi Biotechnology Co., Ltd.) freeze dryer to process the specimen. The freeze‐dried specimen was cut to expose the inside and sputtered with gold to facilitate imaging with SEM (Thermal Field Emission Scanning Electron Microscope, Zeiss, Gemini SEM 300, Germany). In situ confocal microscopy was carried out characterize crack propagation in hydrogels (Zeiss, LSM780).

### Crystallinity and Bond Characterization

In order to characterize the crystallinity of the hydrogel after mechanical training, the hydrogel specimen needs to be immersed in deionized water for 24 h before using the Scientz‐18ND (Ningbo Xinzhi Biotechnology Co., Ltd.) freeze dryer to process the specimen. The crystallinity of the hydrogels was measured by X‐ray diffraction (XRD), small angle X‐ray scattering (SAXS) and differential scanning calorimetry (DSC), respectively.

### Electromechanical Characterization

The trial was made into a dumbbell shape (length: 75 mm, width: 4 mm, thickness: 2 mm) and fixed on an electronic universal testing machine (UTM2102, Shenzhen Sun Technology Co., Ltd.) with a 20 N load cell, and tensile test was performed at 10 mm min^−1^ under displacement control conditions. During the electrical test, the resistance was measured with a resistance scanner (TH2518A, Changzhou Tenhui Electronics Co., Ltd.) by connecting the measurement cable to the leads of the LMTE.

In the cyclic test, considering the failure strain obtained from the tensile test, the maximum strain was set to 50%, the tensile test was carried out at 50 mm min^−1^ under displacement control conditions, and performed 20 000 cyclic tests at a strain of 0–50%. All of the above experiments were carried out in a custom‐built water bath, taking into account the effects of hydrogel drying.

### Water Content Measurement

The water content of the hydrogel was measured by comparing the weights before and after freeze drying. Nitrogen was used to remove excess water on the surface of the hydrogel, and then freeze drying. The weight before (*m*_0_) and after (*m*_a_) freeze‐drying. The water content was obtained as [(*m*_0_) – (*m*_a_)/*m*_0_] × 100%.

### Fracture Test

The hydrogels were cut into rectangular specimens with a length of 38 mm, a width of 8 mm, and a height of 1 mm for fracture testing. An initial gripping distance of 15 mm was used for each pair of samples. All samples had a microstructural arrangement parallel to the height direction. Briefly, for notched samples, a straight line cut ≈2 mm long was made from the middle of the long side of the hydrogel toward the center of the hydrogel and the samples were loaded at a strain rate of 5 mm s^−1^. The critical strain for crack propagation (*ε*_c_) was obtained from the strain at the maximum stress. Paired unnotched specimens were then loaded until *ε* = *ε*_c_. The fracture energy value was obtained by multiplying the area under the stress‐strain curve of the unnotched specimen by the initial grip distance (*H*), *Γ* = *H* $\int_{\varepsilon }^{\varepsilon_{c}}\;$ *σdε*.

### Fatigue Tests

To verify the fatigue resistance of the hydrogels, the single‐notch method was adopted. Cyclic tensile tests were performed using notched samples with an initial crack length (*c*_0_) <1/5 of the sample width (*L*_0_). The crack propagation of the hydrogels was monitored using a digital camera. All stretching cycles are performed continuously. The energy release rate (*G*) was obtained using *G* = 2*kcW*, where, *k* is a function of strain, determined empirically as *k* = 3/$\sqrt{\varepsilon +1}$, *c* is the crack length, and *W* is the strain energy density of an unnotched sample with the same dimensions and stretched to the same strain *ε*.

### Signal Monitoring

For human, LMTE was attached to the volunteer's position and the change in resistance is analyzed by a resistance scanner (TH2518A, Changzhou Tenhui Electronics Co., Ltd.). For in vivo implantation in rat, array hollow cylinders were prepared by a 3D printer (EFL‐BP8601, Suzhou Yongqinquan Intelligent Equipment Co., Ltd), strengthened by the strategy in this paper, and then liquid metal was injected into the hollow cylinders on both sides, and finally LMTE was sutured directly to the ruptured sciatic nerve of the rat. For rat surface monitoring, liquid metal is coated on a strong film and the corresponding signal is obtained by the biological function experimental system (BL‐420s, Chengdu Taimeng Software Co., Ltd). All experiments were conducted under approval from Nanjing Medical University (protocol number: KY20230330‐01‐KS‐01). Volunteers for the author himself. All work involved informed consent from the subjects.

### Cell Culture and Muscle Contraction Simulation

TPSCs were isolated, screened and cultured as suggested in previous studies from Sprague Dawley rats. For muscle contraction simulation, the feasibility test of the hydrogel for muscle contraction simulation was done by the simulator. The training and initial hydrogels were loaded separately and then subjected to 0%‐100% stroke changes with their integrity recorded. Later, 1 × 10^6^ of P2 TPSCs were seeded on hydrogel (XX*XX*XX) for 24 h of culture adaptation. Then in the next four days, ultra‐hydrogels loaded with TPCSs were immobilized on self‐developed muscle simulator fixtures and immersed in phenol red‐free medium (DMEM, gibco; 4% FBS, gibco) for stretching. Different groups were stretched with 0%, 20%, 50%, and 100% deformation to simulate muscle contraction (speed: 0.2 mm s^−1^, 10 cycles time^−1^, 2 times a day). All samples were supplemented with 4% FBS and 1% PS were inoculated at 37 °C in a 5% CO_2_ incubator when they were not simulated. Later at Day 5–14, all hydrogels were placed in a normal culture environment (DMEM, 10%; 10% FBS; 1% PS).

### Adhesion and Proliferation Assays

The cell adhesion morphology was investigated on TPSCs, which had just completed the muscle contraction simulation. Specifically, at Day 4, the culture medium of all 0%, 20%, 50%, 100% groups were cleaned, fixed, and then dehydrated by graded ethanol for SEM observation. As for proliferation assays, CCK‐8 assays were used to detect the growth level of TPSCs every two days during Day 0–10.

### Immunofluorescence Staining of Cells

For the immunofluorescence staining of ITG*β*1 and YAP, at Day 4, TPSCs in different groups were cleaned and fixed, following with Anti‐ITG*β*1 (1:100, Invitrogen) or Anti‐YAP1 antibody (1:500, Abcam). Besides, cell samples for TNMD were on 14th day, which later stained with Anti‐Tenomodulin (1:100, BIOSS). Alexa Fluor 594‐conjugated goat anti‐rat IgG was used as the secondary antibody, with also FITC‐conjugated phalloidin for F‐actin. DAPI containing solution was used to stain the nuclei. All the samples were captured by an inverted fluorescence microscope (Zeiss).

### RT‐PCR Analysis of Cells

The expression levels of some genes in related signal pathways of TPCSs on ultra‐hydrogels activated by muscle contraction simulation were verified by a quantitative real‐time PCR, including *itgb1, yap, taz, mkx, tnmd, co1a1* and *col1a2*. For detection of *itgb1, yap* and *taz*, at Day 4, the TPSCs cultured on the hydrogels of 0%, 20%, 50% were washed and broken for extracting the total RNA, with reverse‐transcribed into complementary DNA using a PrimeScript RT reagent kit. The expression level of *mkx, tnmd, co1a1* and *col1a2* were detected from the samples from each group collected at Day 14. qRT‐PCR was carried out by employing iTaq universal SYBR Green supermix (Applied Biosystems, USA) with the gene‐specific primers listed in Table S2, Supporting Information.

### Western‐Blot

For Western‐blot assay, total protein was first extracted from TPCSs on different muscle contraction simulation groups and quantified by BCA detection. After protein denaturation by thermal cycle, the samples were loaded in the gel with electrophoresis and transferred to PVDF film. Then, the PVDF films were into the primary antibody (primary antibody concentration: COL1 1:1000, YAP 1:3000, *β*‐actin 1:2000, Servicebio). After incubated overnight at 4 °C, the films were incubated by secondary antibody (diluted concentration: 1:5000) following with been washed and luminescence detected.

### Transcriptome Sequencing and Bioinformatics Analysis

Samples of TPSCs from each group at Day 14 were selected for transcriptome analysis. The total RNA was obtained via the mirVana miRNA Isolation Kit (Ambion, USA). Sample integrity was measured with an Agilent 2100 Bioanalyzer (Agilent Technologies, USA). The TruSeq Stranded mRNA LTSample Prep Kit (Illumina, USA) was used to generate the transcriptome sequencing library. Illumina sequencing platform (HiSeqTM) was used for transcription sequencing and 150 bp paired‐end reads were generated. The data were used for further analysis, and the read count value was obtained by bowtie2 and express. The R package DEseq2 was used to screen the differentially expressed genes. Twofold changes with *p*‐value < 0.05 were marked as significant. Then, a series of gene functional enrichment analyses.

### In Vivo Studies

The animal experiments of hydrogel were carried out in accordance with the ISO 10993‐10:2010(E) standard. All procedures for animal experiments followed all ethical guidelines for laboratory animals and were approved by Nanjing Medical University. A total of one rabbit (weight 3 kg) was used in this experiment. In short, the tendon of the rabbit was peeled off, and the trained hydrogel was sutured to the tendon defect through a special surgical thread. And then the wound was sutured and disinfected. The rabbit's leg was immobilized in a cast for one week before the cast was removed. Finally, the rabbit can move freely for a certain period of time to take out the specimen. All experiments were conducted under approval from Nanjing Medical University (protocol number: DWSY‐22056187).

### Histology

Firstly, for histology detection, tissue sectioning of 40‐µm thickness was carried out with the help of a microtome (Leica Biosystems). Hematoxylin‐Eosin (H&E) staining and Masson staining was conducted to perform a histomorphology analysis of tendon regeneration in different phases. Processed tissues were fixed with 4% paraformaldehyde for 24 h, and imbedded in a paraffin. Later, stained sections of tissues were observed under light microscope and images were taken (Olympus). Sirius Red staining was used to study the rebuild collagen in tendons. Samples were treated as the same described above for the de‐paraffinization and re‐dehydration and then stained according to Sirius Red Staining kit. The images of Sirius Red were captured by polarized light scanner (Olympus).

### RT‐PCR Analysis of Tissue

Tendon tissue also was used to obtain the expression levels of genes and proteins by Real‐time fluorescence quantitative (RT‐PCR). For genes expression result, an appropriate amount of sample was transferred to TRIzol (add 1 ml TRIzol per 50–100 mg tissue), in which be mashed with a high‐speed cryogenic tissue grinder. After centrifuged, the upper aqueous phase to a new enzyme‐free EP tube with isopropanol added in. RNA precipitate was carried out from above mixture, and then washed with 75℅ ethanol and stored in DEPC water for later use. RT‐PCR instrument (model QuantStudio TM3, produced by ThermoFisher Instruments Co., Ltd., USA) was used, and the Thermo Scientific PikoReal software (Thermo Company) was used to analyze the CT (Threshold cycle) value of each test sample during the PCR process.

### Immunohistochemistry

The tendon tissue was immunohistochemistry stained with COL1 (Proteintech), COL3 (Proteintech), YAP (BIOSS) and TNMD (BIOSS) to evaluate the tendon differentiation and collagen regeneration. All the slides were captured by an inverted fluorescence microscope (Olympus).

### Statistical Analysis

Data are presented as the means ± standard deviations (S.D). Data from experiments were analyzed with Origin 2018 and GraphPad Prism 8. Statistical analysis was performed with SPSS 22.0 statistical software. One way analysis of variance (ANOVA) followed by Tukey's post hoc test was used for comparisons among multiple groups (*p < 0.05; **p < 0.01; ***p < 0.001).

## Conflict of Interest

The authors declare no conflict of interest.

## Supporting information

Supporting Information

Supplemental Movie 1

Supplemental Movie 2

Supplemental Movie 3

## Data Availability

The data that support the findings of this study are available in the supplementary material of this article.
