# Supplementary material for: Tough Gelatin Hydrogel for Tissue Engineering
Source: Adv Sci (Weinh). 2023 Jun 23;10(24):2301665. doi: 10.1002/advs.202301665 (PMC10460895; doi:10.1002/advs.202301665)
Supplement: Supplementary file 1 — Supporting Information [file ADVS-10-2301665-s003.pdf]

ADVANCED SCIENCE
Open Access

Supporting Information

for *Adv. Sci.*, DOI 10.1002/advs.202301665

Tough Gelatin Hydrogel for Tissue Engineering

Ximin Yuan, Zhou Zhu, Pengcheng Xia, Zhenjia Wang, Xiao Zhao, Xiao Jiang, Tianming Wang, Qing Gao, Jie Xu, Debin Shan, Bin Guo, Qingqiang Yao* and Yong He**

Supporting Information

Tough gelatin hydrogel for tissue engineering

Ximin Yuan[1,2†], Zhou Zhu[3†], Pengcheng Xia[4†], Zhenjia Wang[1,2], Xiao Zhao[4], Xiao Jiang[4], Tianming Wang[4], Qing Gao[5], Jie Xu[1,2*], Debin Shan[1,2], Bin Guo[1,2], Qingqiang Yao[4*], Yong He[5, 6, 7*]

[1] *State Key Laboratory of Advanced Welding and Joining, Harbin Institute of Technology, Harbin, 150001, China.*

[2] *National Innovation Center for Advanced Medical Devices, Shenzhen, 457001, China.*

[3] *State Key Laboratory of Oral Diseases, National Clinical Research Center for Oral Diseases, West China Hospital of Stomatology, Chengdu, 610041, China.*

[4] *Institute of Digital Medicine, Nanjing First Hospital, Nanjing Medical University, Nanjing, 210006, China.*

[5] *State Key Laboratory of Fluid Power and Mechatronic Systems, School of Mechanical Engineering, Zhejiang University, Hangzhou, 310027, China.*

[6] *Key Laboratory of 3D Printing Process and Equipment of Zhejiang Province, College of Mechanical Engineering, Zhejiang University, Hangzhou, 310027, China.*

[7] *Cancer Center, Zhejiang University, Hangzhou, 310058, China.*

[†] *These authors contributed equally to this work: Ximin Yuan, Zhou Zhu, Pengcheng Xia.*

[*] *Correspondence to: Yong He, e-mail: yongqin@zju.edu.cn;*

Jie Xu, e-mail: xjhit@hit.edu.cn;

Qingqiang Yao, e-mail: yaoqingqiang@126.com

Relationship between fatigue threshold and molecular chain

In general, Young's modulus is not the same as stiffness. Young's modulus is a property of material composition and stiffness is a property of structure, namely, modulus is an internal property of a material. For example, for a material in tension or compression, its axial stiffness K = A·E/L, where A is the cross-sectional area, E is the Young's modulus, and L is the length of the material.

Fatigue threshold is estimated by $\Gamma_0 = \alpha\psi\ \sqrt{n} l J/V$[1, 2, 3], where ψ is the volume fraction of polymer. Fatigue threshold Γ_0 is controlled by the chain length n of the same network, $\Gamma_0 \sim \sqrt{n}$. Traditional hydrogels are composed of water molecules and a flexible polymer network. Traditional hydrogels molecular chains are arranged in a disorderly manner. The molecular chain that actually participates in the deformation under the action of the axial force is only part of the length, so it cannot have high fatigue resistance.

Here, we force the molecular chains of conventional hydrogels to align along the axial force direction by means of orientation training, making them into anisotropic structures. The length of its molecular chain along the axial force direction is increased, which in turn increases the breaking threshold several times compared to conventional hydrogels.

Relationship between the maximum elongation and molecular chain

The end distance is the distance from one end of the linear polymer chain to the other end. Because the end distance changes with different molecules and different times, there is no definite value and must be averaged, so the mean square end distance is often used[4, 5, 6]. The ratio of the mean square end distance of fully extended and freely rotating polymer chains determines the maximum elongation λ_{max} of the polymer. The mean square radius of gyration is used for branching *P*. Suppose that the polymer chain contains many chain units, and the mass of each chain unit is m_i. Assuming that the distance from the center of massof the polymer chain to the unit of the *i*-th chain is s_i, which is a vector, the average mass of s_i^2 of all chain units is

$$s^2 = \sum_i m_i s_i^2 \,/\, \sum_i m_i \tag{1}$$

For flexible molecules, the value of s^2 depends on the conformation of the chain. The mean square radius of rotation $\overline{s^2}$ is obtained by averaging s^2 over all possible conformations of the molecular chain. It can be proved that for a Gaussian chain, when the molecular weight is very large, the following relationship exists between the undisturbed mean square terminal distance and the undisturbed mean square radius of rotation

$$\overline{h_0^2} = 6\,\overline{s_0^2} \tag{2}$$

The end distance of a free-associating chain consisting of n bonds should be the vector sum of the individual bond lengths

$$\overline{h_{a,b}^2} = \sum_{a=1}^{n} \sum_{b=1}^{n} \overline{\overrightarrow{l_a} \cdot \overrightarrow{L_b}} \tag{3}$$

where f, j is the free spinning chain.

$$\overline{h_{a,b}^2} = \sum_{a=1}^{n} \sum_{b=1}^{n} \overline{\overrightarrow{l_a} \cdot \overrightarrow{l_b}} = \begin{vmatrix} \overline{\overrightarrow{l_1} \cdot \overrightarrow{l_1}} + \overline{\overrightarrow{l_1} \cdot \overrightarrow{l_2}} + \cdots + \overline{\overrightarrow{l_1} \cdot \overrightarrow{l_n}} \\ +\overline{\overrightarrow{l_2} \cdot \overrightarrow{l_1}} + \overline{\overrightarrow{l_2} \cdot \overrightarrow{l_2}} + \cdots + \overline{\overrightarrow{l_2} \cdot \overrightarrow{l_n}} \\ \cdots\cdots\cdots\cdots\cdots\cdots\cdots\cdots\cdots \\ +\overline{\overrightarrow{l_n} \cdot \overrightarrow{l_1}} + \overline{\overrightarrow{l_n} \cdot \overrightarrow{l_2}} + \cdots + \overline{\overrightarrow{l_n} \cdot \overrightarrow{l_n}} \end{vmatrix} \tag{4}$$

Diagonal terms $\overline{\overrightarrow{l_a} \cdot \overrightarrow{l_b}} = l^2$, a total of *n* terms;

Adjacent diagonal items $\overline{\overrightarrow{l_a} \cdot \overrightarrow{l_{b+1}}} = l(-\cos\theta)l = l^2\,(-\cos\theta)$, a total of 2(n-1) items;

Thus, $\overline{\overrightarrow{l_a} \cdot \overrightarrow{l_{b+m}}} = l^2(-\cos\theta)^m$, a total of 2(n-m) items;

The above results are brought into (4)

$$\overline{h_{a,b}^2} = l^2[n + 2(n-1)(-\cos\theta) + 2(n-2)(-\cos\theta)^2 + \cdots + 2(-\cos\theta)^{n-1}]$$

$$= nl^2\left\{\left(\frac{1-\cos\theta}{1+\cos\theta} + \frac{2\cos\theta}{n}\right)\left(\frac{1-(-\cos\theta)^n}{(1+\cos\theta)^2}\right)\right\} \tag{5}$$

because $n \to \infty$,

$$\overline{h_{a,b}^2} = nl^2\,\frac{1-\cos\theta}{1+\cos\theta} \tag{6}$$

For single-network hydrogels, the steric effect is not considered, the mean square end distance of fully extended is

$$\overline{h_{a,b}^2} = 2nl^2 \tag{7}$$

The mean square end distance of freely rotating is

$$\overline{h_{max}^2} = n^2 l^2 \frac{1 - \cos\theta}{2} \approx \frac{2}{3} n^2 l^2$$

The maximum elongation λ_{max} of the polymer is

$$\lambda_{max} = \frac{\overline{h_{max}^2}}{\overline{h_{a,b}^2}} = n \cdot \frac{1 + \cos\theta}{2} \approx \frac{n}{3}$$

So $\lambda_{max} \sim n$.

Due to $\Gamma_0 \sim \sqrt{n}$, $\lambda_{max} \sim n$, Γ_0 and λ_{max} all increase as n increases. Therefore, high fatigue threshold materials are often accompanied by high maximum elongation.

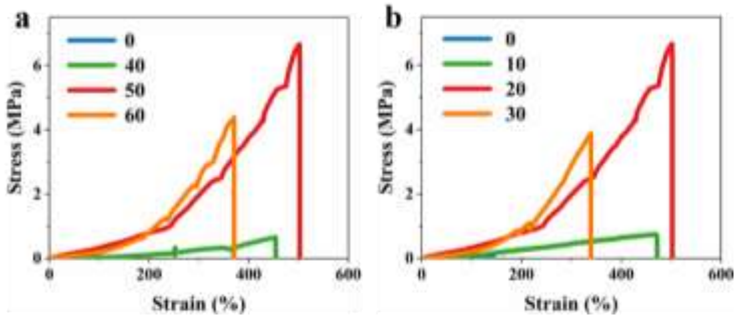

Figure S1. Mechanical properties of hydrogels. (a) Mechanical properties of hydrogel as a function of number of training times. (b) Mechanical properties of hydrogel as a function of salt concentration.

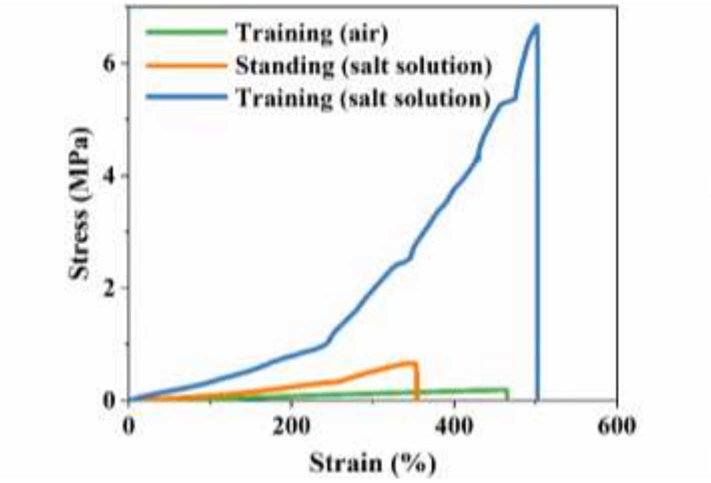

Figure S2. Mechanical properties of hydrogels with different treatment processes.

To further demonstrate the synergistic effect of stretching and salt solution (SO_4^{2-}), we stretched the same size hydrogels in air and immersed them in salt solution (controlling one variable and keeping the other conditions unchanged). It can be clearly found that the hydrogel stretched only in air has a significant increase in strain and a weaker increase in stress. In contrast, hydrogels immersed only in saline solution had a significant increase in stress and a weaker increase in strain. It was further confirmed that it was precisely because of the synergistic effect of stretching and salt solution that the stress and strain of the hydrogel were significantly increased.

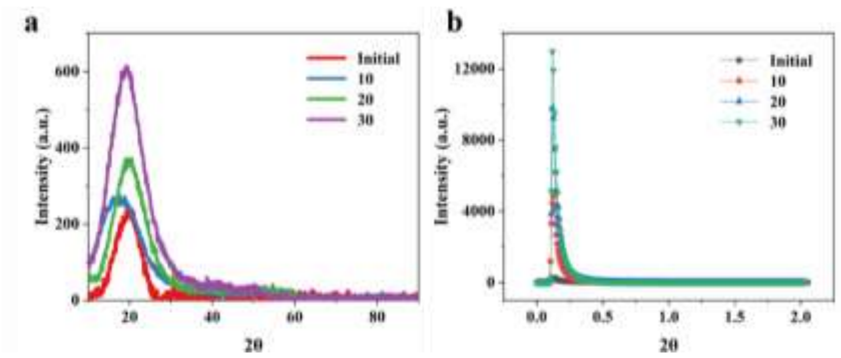

Figure S3. Crystal structure analysis of tough hydrogel. a XRD, b SAXS.

The XRD and SAXS characterizations showed that the crystallinity of the hydrogel increased correspondingly with the increase of training times. And the results of XRD and SAXS characterization were roughly the same, which further proves the consistency of the data. This result was consistent with the DSC characterization results.

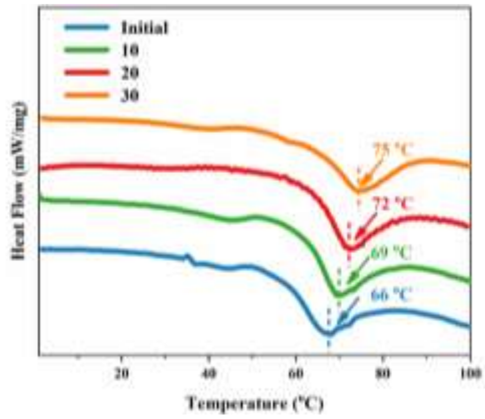

Figure S4. DSC measurement of hydrogel.

According to the DSC characterization, the melting point of the hydrogel gradually increases with the increase of training times, which implies that the crystallinity of the hydrogel increases with the increase of training times.

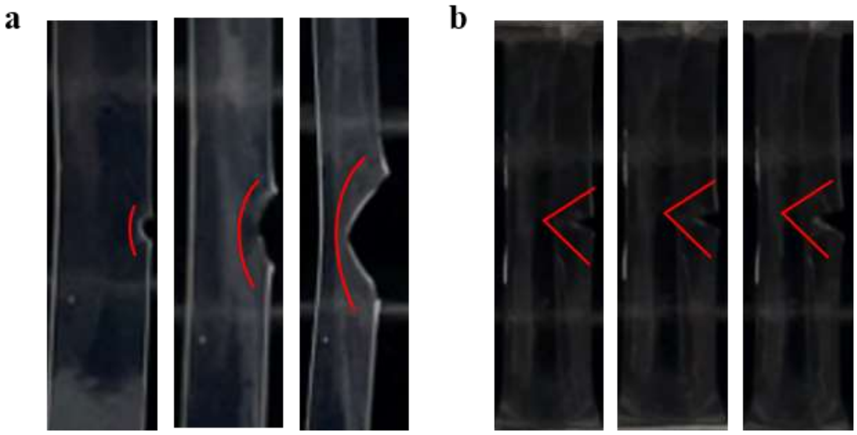

Figure S5. Optical images of unilateral axial tensile notch crack. a: gelatin-based tough hydrogel, b: initial hydrogel.

It can be clearly seen from the optical images that the hydrogel after training has excellent resistance to crack propagation, and the notch was arc-shaped, suggesting that the trained hydrogel has the ability to passivate cracks. However, conventional hydrogels do not have the ability to resist crack propagation, and the notch was sharp.

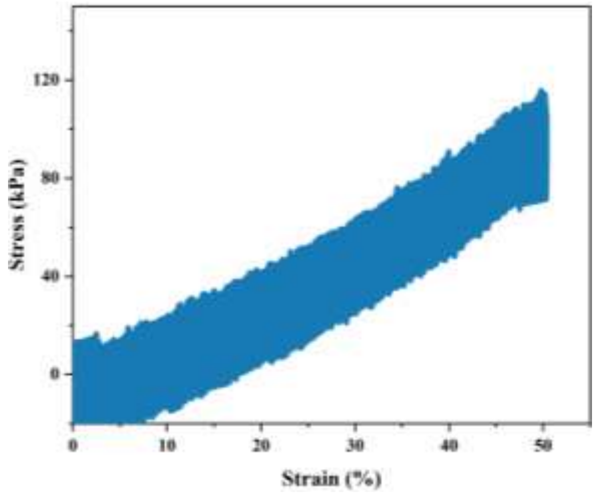

Figure S6. Tough notched hydrogel cycles axially 1000 times.

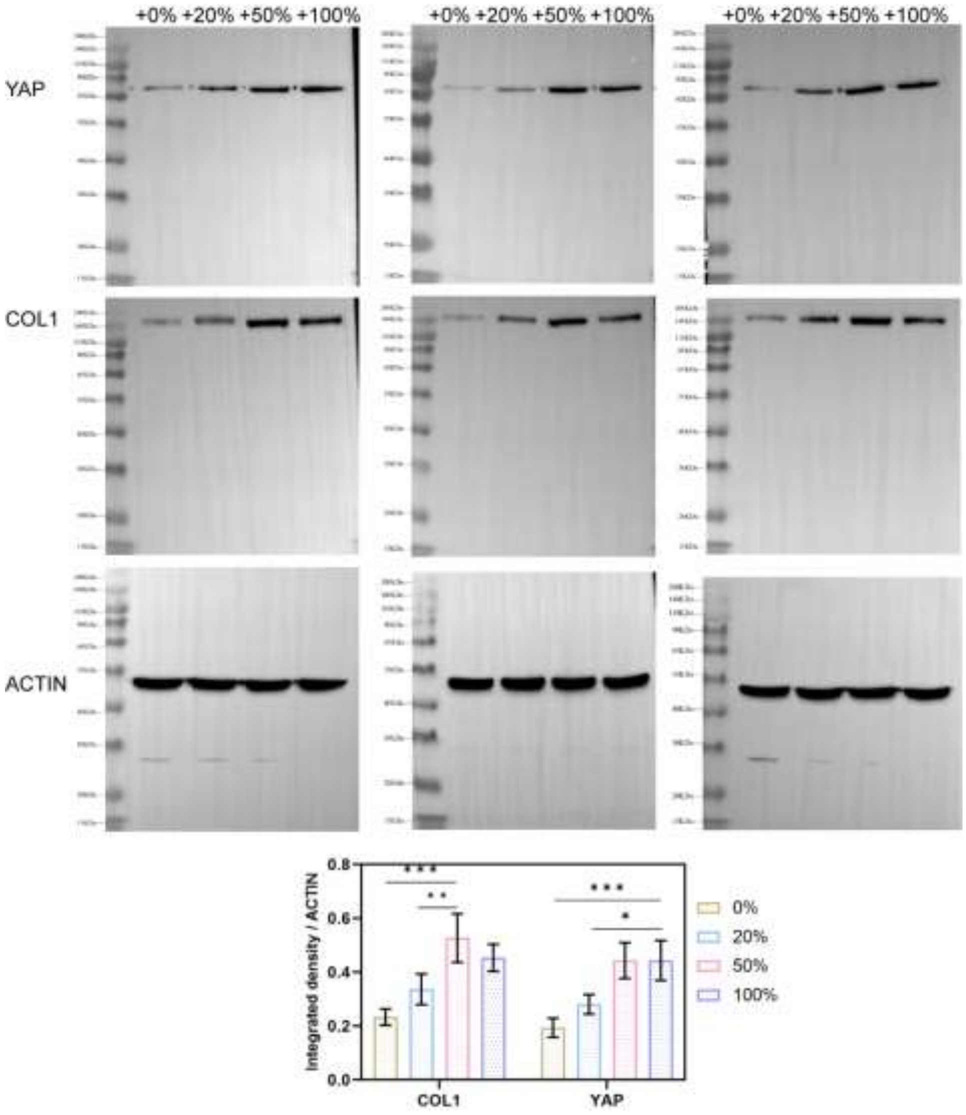

Figure S7. Complete Western blot images and semi quantitative results. **P* < 0.05; ***P* < 0.01; ****P* < 0.001. n = 3.

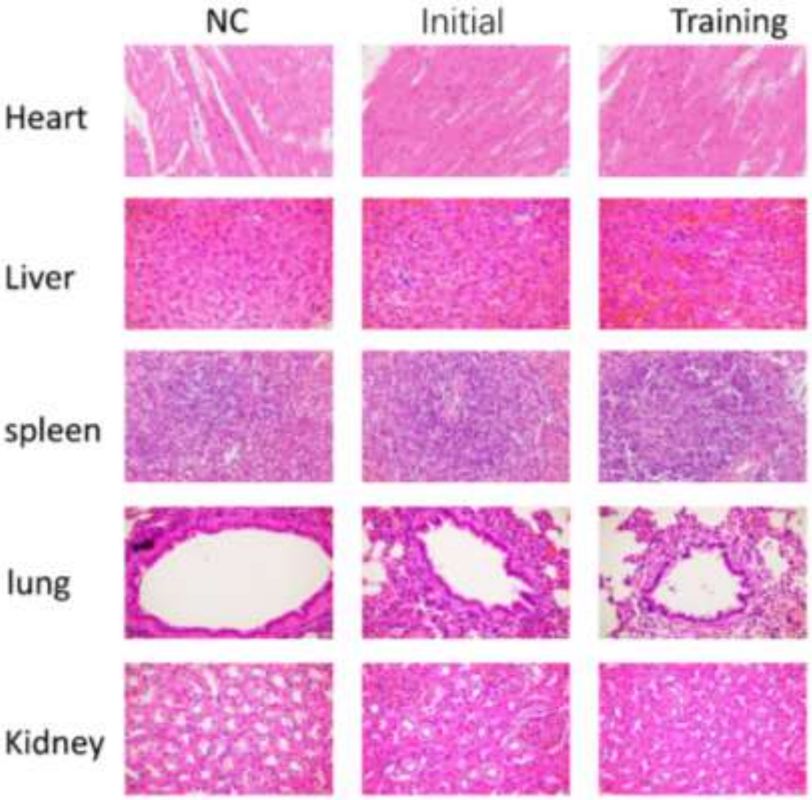

Figure S8. H&E image of main organs of rats after implantation of tough hydrogel.

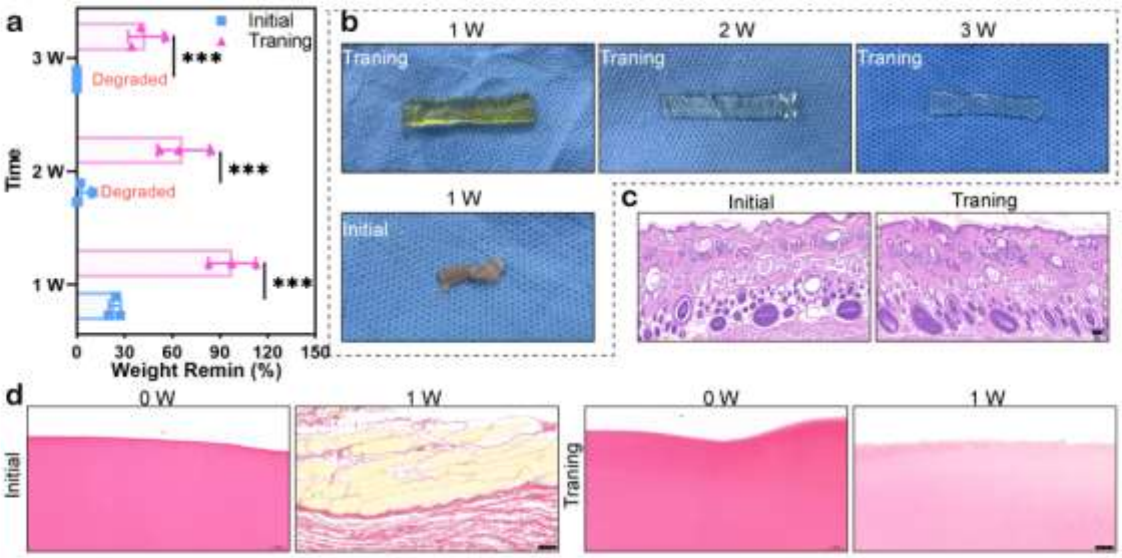

Figure S9. a. In vivo degradation of GBTH in rats after 1 to 3 weeks. n = 3; $***P < 0.001$. **b.** Image of degraded hydrogel. **c.** H&E staining of skin implanted with hydrogels. **d.** Sirius red staining of hydrogels at 0W and 1W. All scale bar: 100 μm.

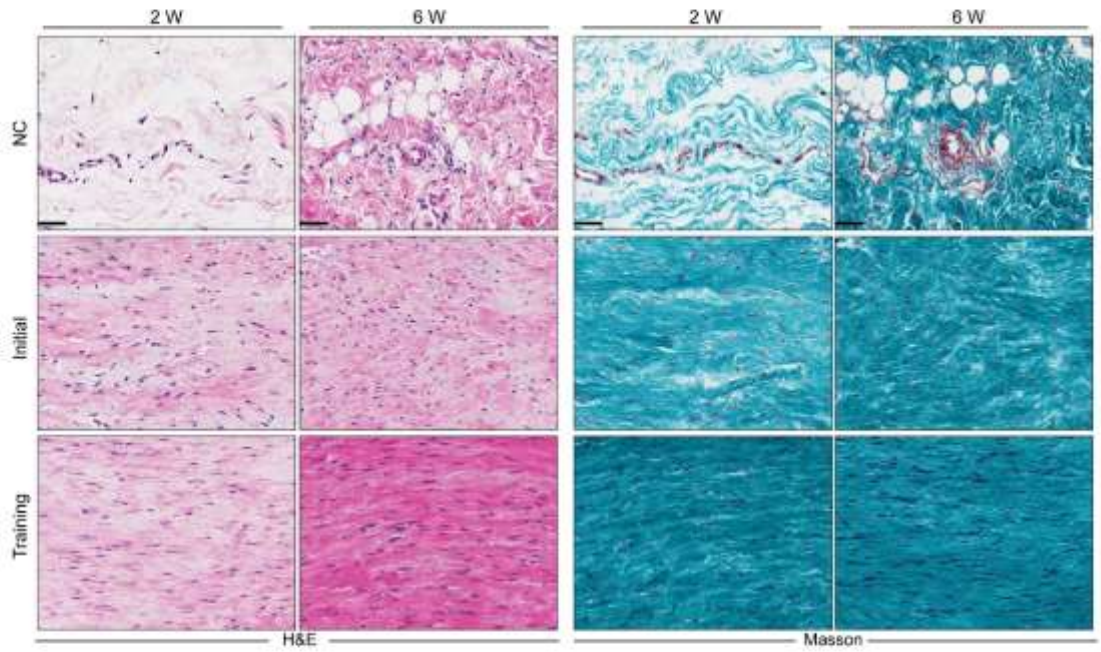

Figure S10. H&E and Masson staining of tendon tissues at two and six weeks. n = 3. All scale bars indicate 200 μm.

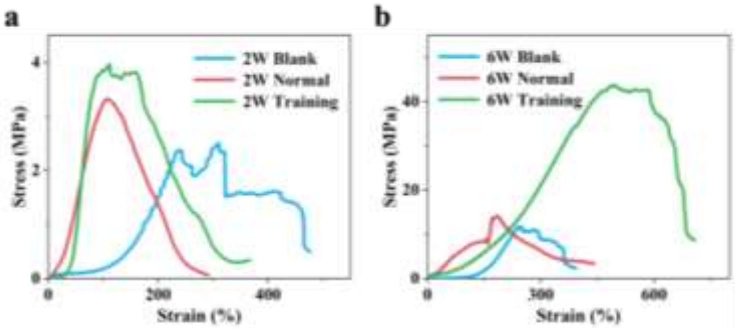

Figure S11. Mechanical properties of rabbit tendons at different times. a: 2 Week (W), b: 6 W.

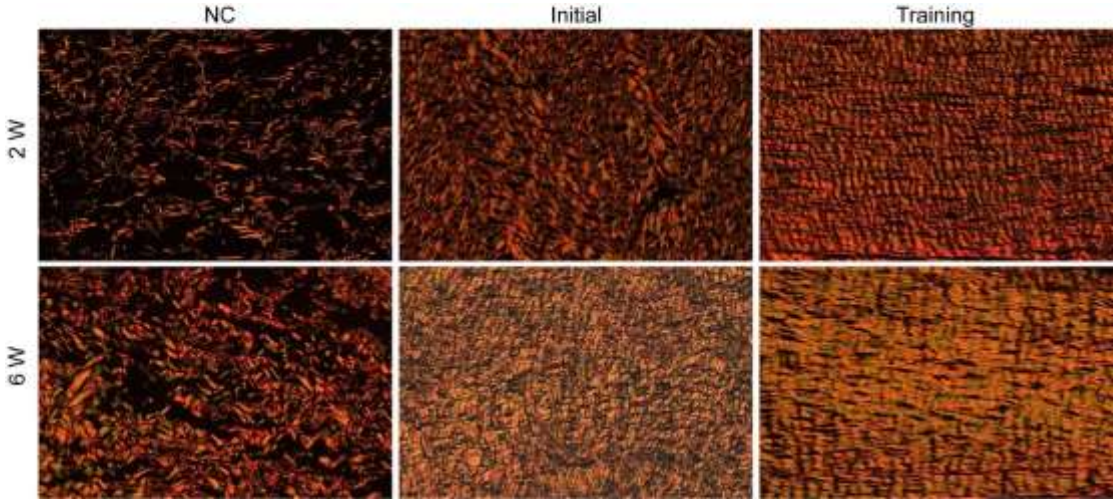

Figure S12. Polarized observation results of Sirius red staining at 2W and 6W.

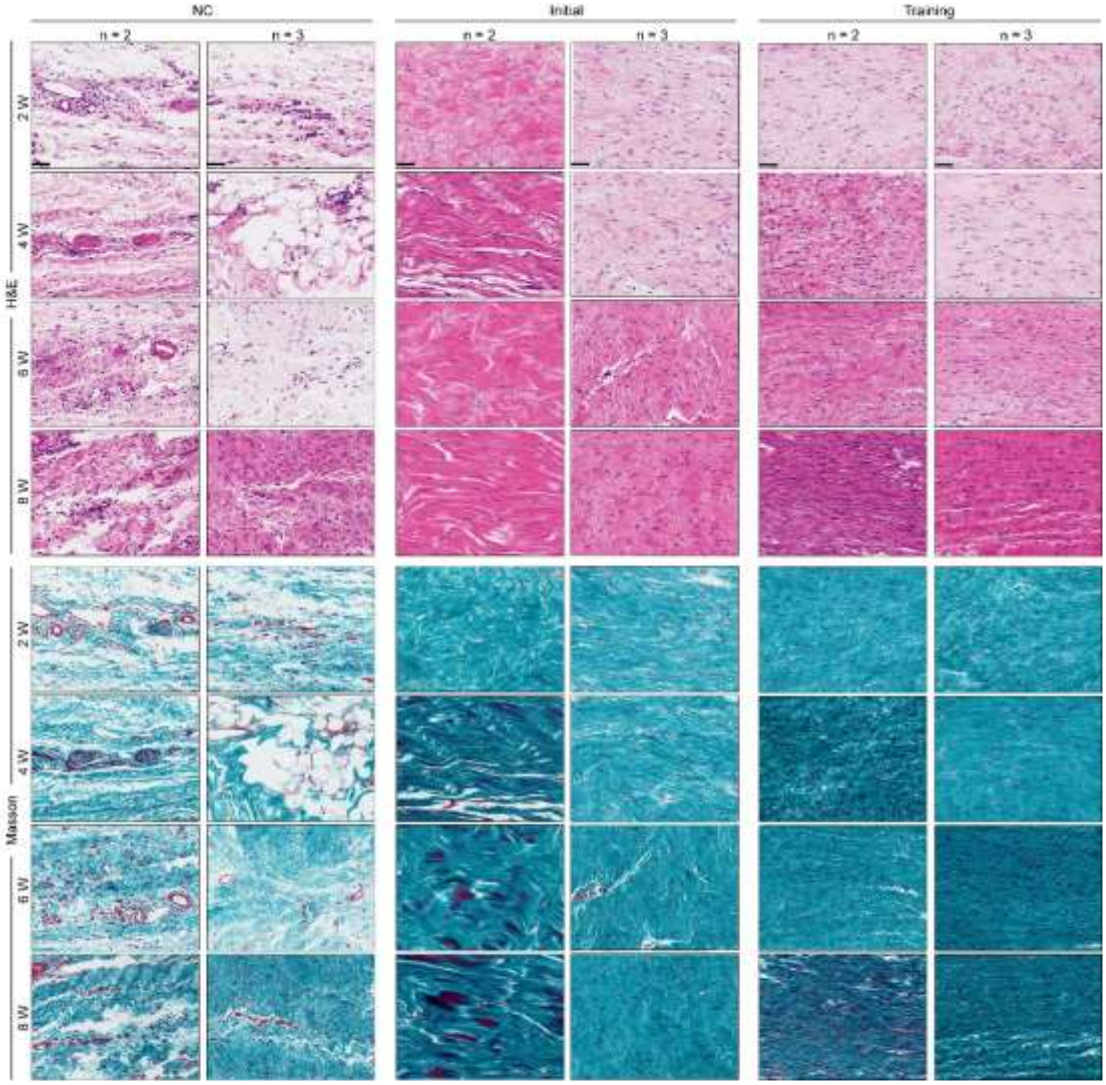

Figure S13. Stained images of all remaining samples.

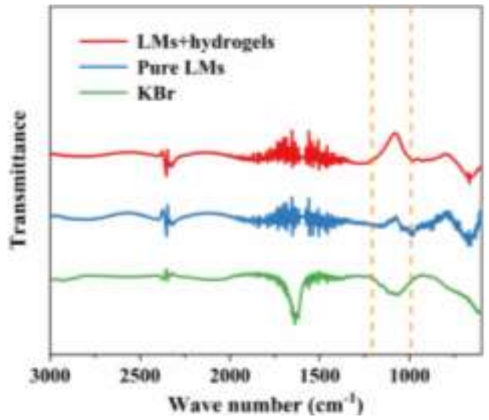

Figure S14. FTIR measurements of LMs.

From the FTIR results, it can be seen that the LMs may be anchored on the surface of the hydrogel, and the LMs particles fuse with each other to reduce the resistance during the stretching process. Therefore, LMTE still maintains stable deformation electrical responsiveness under large deformations.

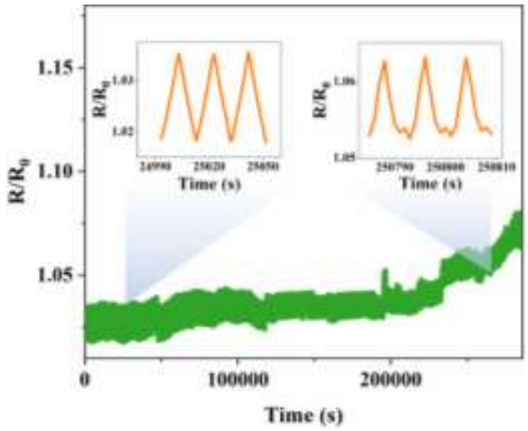

Figure S15. Resistance changes for 20,000 LMTE cycles.

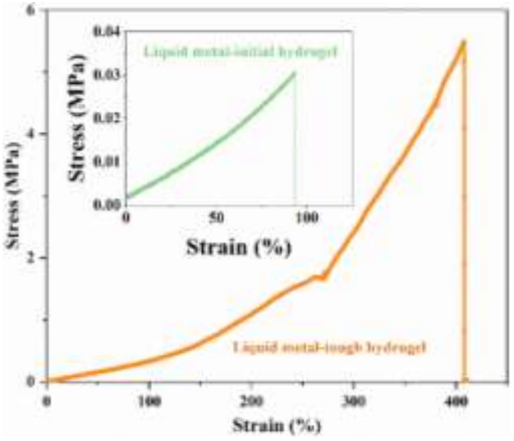

Figure S16. Mechanical properties of liquid metal based tough hydrogel electrons. The inset is the mechanical properties of liquid metal based initial hydrogel electrons.

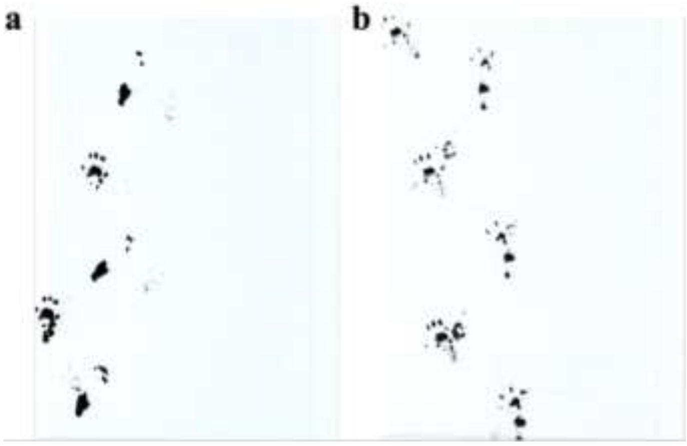

Figure S17. Rat footprint diagram. a: Blank group; b: Repair the group (2 months).

The left foot is the control group and the right foot is the experimental group. The right foot (severed sciatica) in the blank group can be clearly seen as atrophied and the left foot diastolic. However, when we implanted the conductive scaffold into the left foot (severed sciatic nerve) for 2 months, we could find that the right foot was diastolic to a certain extent, indicating that the conductive scaffold had a certain repair effect.

Table S1. Comparison between different methods.

	Stress (MPa)	Cycles	Water (%)	Γ (KJ/m^2)	G (KJ/m^2)	Component	Bio-compatibility	Bionic structure	Time (h)
**This work**	**6.67**	**20000**	**80**	**297**	**28**	**Gelatin**	□	□	**0.5 h**
**[30]**	10	100	75	131	10	PVA	—	□	120 h
**[43]**	1	50000	—	4.6	0.4	PAAm + PAA	—	—	24 h
**[36]**	8.4	5000	—	0.8	0.6	PVA + Triton X-100	—	□	3 h
**[40]**	4.7	100	47	—	—	Silk + $CaCl_2$	—	□	0.5 h
**[42]**	0.9	—	80	2.8	—	PNaAMPS + MBAA+ $(NH_4)_2Fe(SO_4)_2$	—	—	120 h
**[47]**	0.15	1500	—	11.4	—	Alg + PAAm	—	—	2.5 h
**[44]**	0.9	—	—	—	4	PEG + calcium phosphate + collagen	—	□	2 h
**[25]**	—	—	—	—	1.3	Alg + PAAM + chitosan + Ca^{2+}	□	—	24 h
**[26]**	25.7	—	—	—	—	Gelatin + HA PCL	□	□	240 h
**[27]**	19	—	—	—	—	CS Nanowire + Alg + Ca^{2+}	□	□	36 h

Table S2. Primers for Quantitative Real-Time PCR.

	Name	Primer	Sequence (5’ -3’)
	YAP-1	Forward	GACAACAACATGGCAGGACCC
	YAP-1	Reverse	TGAGGCAGAGTTCATCAGCGT
	COL1A1	Forward	CGTGGAAACCTGATGTATGCTTG
	COL1A1	Reverse	CCTATGACTTCTGCGTCTGGTGA
	COL1A2	Forward	ACCCAGAGTGGAAGAGCGATTA
Rat	*COL1A2*	Reverse	TGGATGCAGGTTTCACCAGTAGA
	ITGB1	Forward	TCAACTGCGATAGGTCCAACG
	ITGB1	Reverse	CACTGAACACATTCTTTATGCTCTG
	TAZ	Forward	GGCATGTTGGAATGAATGATGT
	TAZ	Reverse	AGCACAGGGAGTGTACTGAAGG
	TNMD	Forward	GTTTTGGGGGAGCAAACACT
	TNMD	Reverse	ATGTTTCATCGGTGCCATTT
	GAPDH	Forward	CTGGAGAAACCTGCCAAGTATG
	GAPDH	Reverse	GGTGGAAGAATGGGAGTTGCT

References

1. Z. P. Zhou, D. Y. Yan, *Macromol. Theory Simul.* **2003**, *6*, 597.
2. R. J. Rubin, *J. Chem. Phys.* **1965**, *43*, 237.
3. V. A. Nguyen, S. Shafieezadeh-Abadeh, D. Kuhn, P. M. Esfahani, *Math. Oper. Res.* **2021**, *11*, 1176.
